# Bandgap reduction of photocatalytic TiO_2_ nanotube by Cu doping

**DOI:** 10.1038/s41598-018-32130-w

**Published:** 2018-09-21

**Authors:** S. Khajoei Gharaei, M. Abbasnejad, Ryo Maezono

**Affiliations:** 1 Faculty of Physics, Shahid Bahonar University of Kerman, Kerman, Iran; 2 School of Information Science, JAIST, Asahidai 1-1, Nomi, Ishikawa, 923-1292 Japan; 3 Computational Engineering Applications Unit, RIKEN, 2-1 Hirosawa, Wako, Saitama 351-0198 Japan

## Abstract

We performed the electronic structure calculations of Cu-doped TiO_2_ nanotubes by using density functional theory aided by the Hubbard correction (DFT + *U*). Relative positions of the sub-bands due to the dopants in the band diagram are examined to see if they are properly located within the redox interval. The doping is found to tune the material to be a possible candidate for the photocatalyst by making the bandgap accommodated within the visible and infrared range of the solar spectrum. Among several possibilities of the dopant positions, we found that only the case with the dopant located at the center of nanotube seems preventing from electron-hole recombinations to achieve desired photocatalytic activity with *n*-type behavior.

## Introduction

TiO_2_ is a semiconductor with varoius polymorphs forming naturally or under high pressure and temperature, such as anatase, rutile and brookite or high pressure cotunnite and fluorite structures^1–4^, as representative phases. Its wider applications include sensors, photovoltaic and lithium batteries due to its exclusive photoelectronic properties, high chemical stability, low cost, and low environmental impacts^5–9^. It has a potential for controlling the environmental pollution, and for converting/storing energies. Its wide bandgap (3.2 eV) is, however, one of the matter hampering the improvements for more efficiency of devices working under the wide range of solar spectrum. So far, a lot of work has been done to investigate possible ways to reduce the bandgap^10–14^. Fabricating it in nanostructures is known to be one of the promising way by increasing the ratio of the surface to the volume. TiO_2_ nanotubes (TiO_2_-NTs) have therefore attracted a lot of attentions in both experimental and theoretical researches investigating their unique optical and chemical properties due to high photocatalytic activity, strong oxidizing agents, and non-toxicity^15^.

However, it is reported that low-dimensional TiO_2_ nanostructures tend to have the bandgap greater than 3.0 eV due to the quantum confinement effects^16,17^, getting rather toward less efficiency in the visible range of the solar spectrum. To make the bandgap back to be reduced less than 3.0 eV, the doping of impurities to the nanotubes is reported as a promising way^18–20^, which chemical stability is experimentally confirmed as well^16^. Cu doping has especially attracted its interest as a good candidate both experimentally and theoretically due to its high abundance with low cost availability^21,22^. In this work, we investigated such a possibility to get properly tuned bandgap by using *ab initio* electronic structure analysis.

To get a plausible model, both in the sense of computationally and geometrically, we constructed it carefully, step by step from a bulk to a pristine tube and to a Cu-doped tube: To justify our computational model (DFT + *U*), we carefully checked first whether the introduced *U* properly reproduced the available experimental values. After fixing the *U* parameter, we constructed a geometry model for the TiO_2_-NT by rolling up a possible surface cut from an anatase bulk. The bandgap evaluated for the pristine tube is confirmed to be consistent with those from other available reports^15,23,24^. Several possibilities of the Cu doping are examined over the cases with a dopant inserted inside the tube, attached on the surface of the tube, substituting titanium (Ti) site with and without an Oxygen (O) vacancy nearby. To the extent of the justification of our model (the limited system size, and the reliability within DFT), the case with a Cu dopant located at the center of nanotube is predicted to realize the reduced bandgap with the recombinations most unlikely to occur. The present result would support the ability of Cu-doped TiO_2_-NT as a candidate of photocatalytic materials, as reported experimentally by Momeni *et al*.^25^, showing that their nanotube improves the photocatalytic behavior.

## Results

All calculations were performed in the framework of DFT (density functional theory)^26^ based on the plane wave and the pseudopotential method. Detailed descriptions for the method are given in the latter section. We used DFT + *U* scheme^27^ since Ti in our system has half-filled *d* orbitals, for which the scheme is widely accepted as a reasonable choice. Since the choice of the Hubbard parameter (*U*) strongly affects the reliability of predictions^28^, we have to calibrate it carefully taking some appropriate reference system. We took the anatase TiO_2_ bulk for the reference because the phase has the highest photocatalytic activity among the other phases being appropriate for the purpose of the present study. Our choice, *U* = 4.2 eV for Ti, gives a reasonable coincidence with experimental values, as summarized in Table 1.

**Table 1:** The calculated lattice parameters (*a* and *c*), relaxed volume (*V*_0_), electronic band gap (*E*_*g*_), bulk modulus (*B*_0_) and its derivative (${B^{\prime} }_{0}$) of anatase TiO_2_ bulk structure compared to the other experimental and theoretical values.

Method	*a*	*c*	*V*_0_	*E*_*g*_	*B*_0_	${{\boldsymbol{B}}{\boldsymbol{^{\prime} }}}_{{\bf{0}}}$
	(Å)	(Å)	(Å^3^/TiO_2_)	(eV)	(GPa)	
GGA-PBE + *U* [present work]	3.893	9.663	36.61	2.58	167	4.0
GGA-PBE [present work]	3.796	9.579	34.51	2.10	177	4.8
GGA-PBE + *U*^11^	3.834	9.632	35.40	2.00	—	—
GGA-PBE^58^	3.800	9.670	34.91	2.25	—	—
PBE^59^	3.786	9.867	35.36	—	—	—
LDA^60^	3.758	9.495	33.52	—	194	—
Exp.^20^	3.784	9.507	34.03	3.18	—	—
Exp.^61^	3.785	9.511	34.07	—	179	4.5

**Figure 1:**
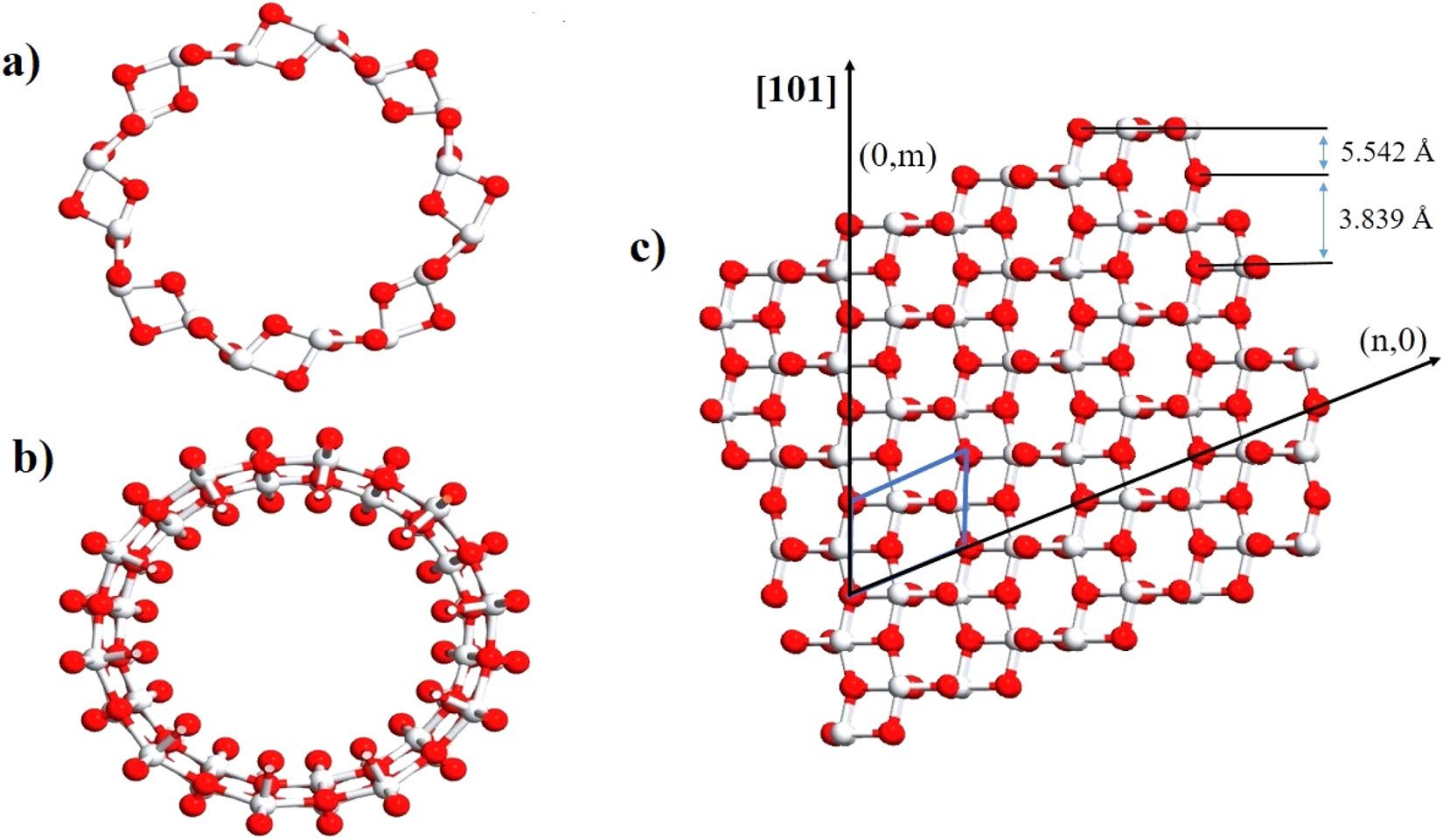
A general view of (**a**) (0, *m*), (**b**) (*n*, 0) TiO_2_-NT, and (**c**) (101) surface of TiO_2_. The red and white spheres represent the O and Ti atoms, respectively.

Having fixed *U* for Ti, we performed the calculations for TiO_2_-NTs. The structure of the one-dimensional single-wall nanotube is formed by rolling up a possible surface of anatase TiO_2_ bulk^23^. Though both (001) and (101) surfaces are known to be found in natural crystals, ultrathin (001) surfaces are reported being not feasible to form a tube due to its surface reconstructions^15^. We therefore took the (101) surface taken from the bulk (Table 1) to construct our nanotubes. Fig. 1 shows the geometry of the nanotube with different chiralities, (*n*, 0) and (0, *m*). We considered only (0, *m*) because it is reported to realize less strain compared to (*n*, 0) under the same radius^15,24^. To make sure whether our nanotube structure could describe realistic samples to some extent, we examined the dependence of the calculated properties on *m*^29^, as tabulated in Table 2. All the computational details are given in Sec. ‘Computational Details’.

**Table 2:** The diameter (*D*) and thickness (*T*) of the pristine (0, *m*)-TiO_2_-NTs before and after relaxation. *N* is the number of atoms in the structure, and *E*_*form*_ and *E*_*g*_ are the formation energy and the electronic bandgap of the TiO_2_-NTs, respectively. For Ti-O bond lengths, each Ti atom is bonded to four O atoms as in Fig. 5 (left one). The O atoms are marked with numbers 1 to 4. For pristine NT, it is tractable upto *m* = 6, while when a Cu-dopant is attached, *m* = 3 gets to be the tractable upper limit (see text).

nanotube	*N*	*D*(Å)		*T*(Å)		*E*_*form*_ (eV)	*E*_*g*_ (eV)	
		Before relaxation	After relaxation	Before relaxation	After relaxation			
(0,3)	36	7.798	7.536	2.485	2.474	0.661	3.26	
(0,4)	48	10.683	10.368	2.488	2.478	0.626	3.46	
(0,5)	60	14.307	14.193	2.489	2.479	0.604	3.55	
(0,6)	72	17.488	17.395	2.490	2.480	0.589	3.61	
**nanotube**	**Ti-O (Å)**							
	**Before relaxation**				**After relaxation**			
	**Ti-O**_**1**_	**Ti-O**_**2**_	**Ti-O**_**3**_	**Ti-O**_**4**_	**Ti-O**_**1**_	**Ti-O**_**2**_	**Ti-O**_**3**_	**Ti-O**_**4**_
(0,3)	1.958	2.000	1.708	1.961	1.988	1.864	1.836	2.070
(0,4)	2.014	1.925	2.208	1.968	2.019	1.810	1.915	2.007
(0,5)	1.967	2.168	1.933	2.013	2.012	1.931	1.800	2.010
(0,6)	1.961	1.984	1.858	1.985	1.984	1.792	1.900	2.076

The evaluated dependence of the bandgap agrees with those by the other reported works in the sense that the semiconducting behavior is kept regardless of their chirality^30^ while the increase of bandgap is observed as the tube diameter increases. From the dependence on *m*, we can estimate the bandgap to be compared with the experimental sample size. The estimation is fairly in consistent with the available experimental values, ranging 3.15–3.42 eV^25,31–37^. The dependence of the formation energy agrees with those reported in preceding works^15,23,24^ as well, getting decreased as the diameter increases. The dependence of the bandgap on *m* might be related with the reported dependence of the photocatalytic activity on the system size^38^. A preceding theoretical analysis by a kinetic model^38^ reports that the photocatalytic activity initially increases and then decreases as the diameter (*D*) and the wall thickness (*T*) increases, being attributed to the change in the light absorption, the surface area, and the reactant transport. Provided that the initial increase would be extrinsic due to some sample quality issues, the trend shown in Table 2 might properly be capturing the asymptotic decrease of the activity depending on *T* and *D*, regardless of the mismatch in the range of *m* between the experimental system size and our feasible range of it.

**Figure 2:**
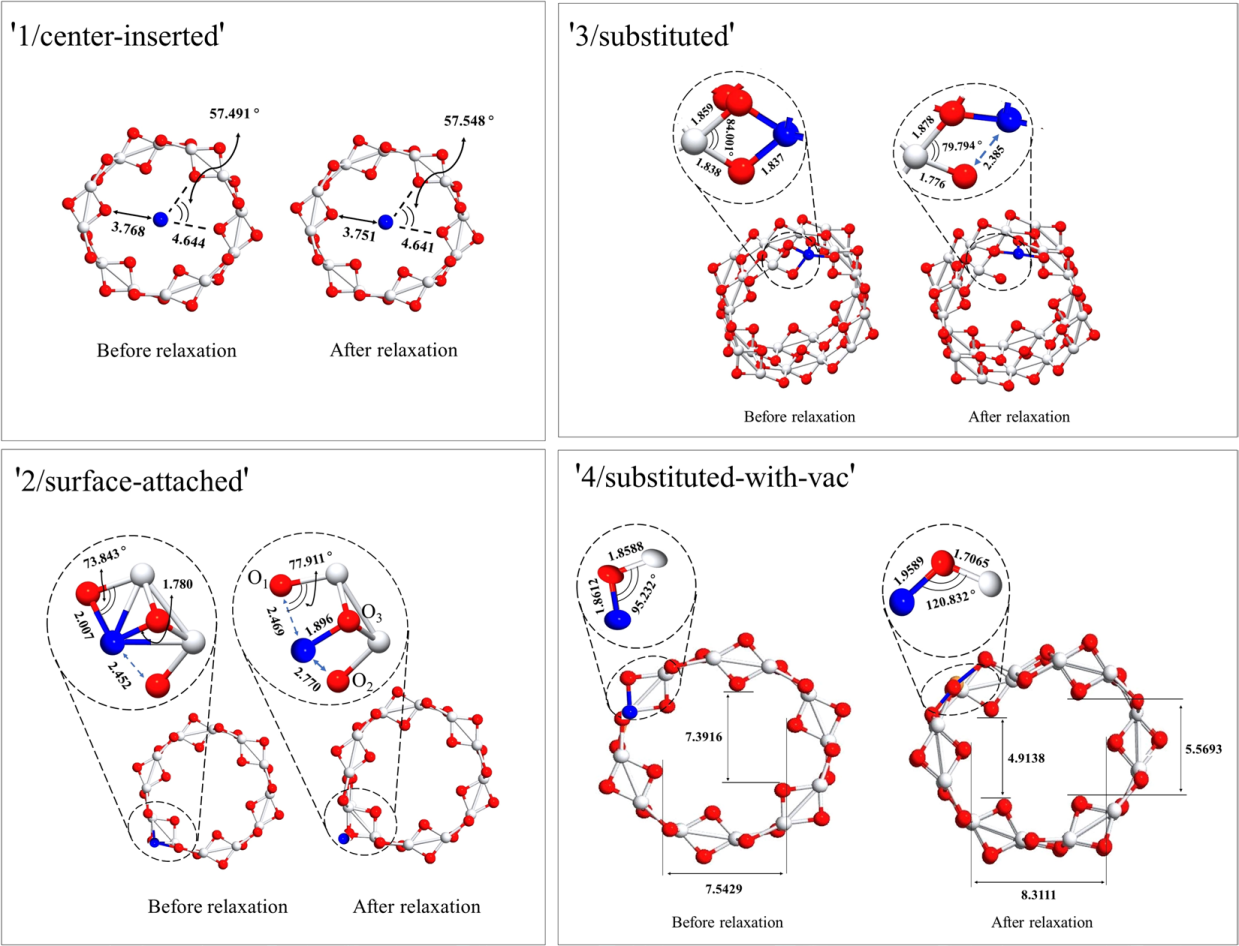
Four cases considered for the Cu-doping model. White, red and blue spheres represent Ti, O and Cu atoms, respectively. All distances are in unit of Angstrom.

**Figure 3:**
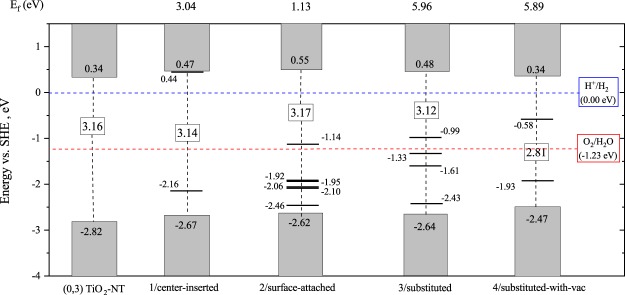
Band-lineup diagram for pure and Cu-doped TiO_2_-NTs in different configurations. The blue and red dotted line are the redox potentials, ${\varepsilon }_{{H}^{+}/{H}_{2}}$ and ${\varepsilon }_{{O}_{2}/{H}_{2}O}$, respectively. The zero of the energy scale corresponds to the potential of the standard hydrogen electrode SHE (${\varepsilon }_{{H}^{+}/{H}_{2}}$), which explains the difference to the one-electron levels in respect to vacuum zero as presented in Table 3. Solid horizontal lines describe *ε*_*HOIL*_ and *ε*_*LUIL*_ levels, below and above the redox potential ${\varepsilon }_{{H}^{+}/{H}_{2}}$, respectively. The rectangles correspond to the difference between the conduction and the valence bands. *E*_*f*_ values shown at the top are the formation energies of the proposed structures.

For modeling the Cu-doping, we considered four possibilities as shown in Fig. 2, the absorption of a Cu atom located at the center of the tube (‘1/center-inserted’), at the surface of the tube (‘2/surface-attached’), and the substitution by a Cu replacing a Ti site without (‘3/substituted’) and with an Oxygen vacancy (‘4/substituted-with-vac’). We considered the substitution cases because the radius of Cu^2 +^ is near to that of Ti^4 +^, being likely to replace the latter. For the O vacancy, it is reported to improve the optical absorption and the photocatalytic activity^39,40^. It is also reported that the addition of Cu impurities tends to create O vacancies for the charge compensation, making the optical absorption extending toward the visible-light range^22^. For the sake of tractability, we used *m* = 3 for the system size to investigate the doping case. Calculations beyond *m* = 3 are intractable at the present computational power even using a large commercial supercomputer, as described in Sec. ‘Computational Details’. We ought to note that the present results, especially the predictions given in Fig. 3, are restrictive to the extent of the system size here. Photoexcitation phenomena include many-body effects which are known to be critically affected by the finite size errors^41^. The *U* parameter was also introduced for Cu being 5.2 eV taken from the other reports^42^. The value is reported to reproduce the features of experimental *X*-ray photoemission spectroscopies for Cu_2_O^42^. We considered the magnetic polarizations on Cu because more structural stability is found to be achieved by allowing the polarizations.

To get proper photocatalytic functions, it is known^43^ for the candidate materials to satisfy the inequalities:

1 $${\varepsilon }_{{\rm{VB}}} < {\varepsilon }_{{\rm{HOIL}}} < {\varepsilon }_{{{\rm{O}}}_{2}/{{\rm{H}}}_{2}{\rm{O}}} < {\varepsilon }_{{{\rm{H}}}^{+}/{{\rm{H}}}_{2}} < {\varepsilon }_{{\rm{LUIL}}} < {\varepsilon }_{{\rm{CB}}},$$

where *ε*_VB_, *ε*_CB_, *ε*_HOIL_ and *ε*_LUIL_ denote the valance band top, the conduction band bottom, the highest occupied, and the lowest unoccupied impurity levels, respectively. A band-lineup diagram, as shown in Fig. 3, is used as a common way to distinguish the capabilities of materials for the photocatalytic functions. Taking the vacuum levels, given in Table 3, as the common reference level, four cases of Cu-doping are compared on the figure, with the oxidation and the reduction potential level of the water, marked ${\varepsilon }_{{{\rm{O}}}_{2}/{{\rm{H}}}_{2}{\rm{O}}}$ and ${\varepsilon }_{{{\rm{H}}}^{+}/{{\rm{H}}}_{2}}$, respectively.

**Table 3:** Energetics required for the band-lineup diagram (Fig. 3). Units are all given in [eV].

Structure	Fermi Level	Vacuum Level	Work Function
Pristine (0,3) TiO_2_ NT	−4.275	1.698	5.973
1/center-inserted	−2.144	1.734	3.878
2/surface-attached	−2.892	1.711	4.603
3/substituted	−4.519	1.682	6.201
4/substituted-with-vac	−4.559	1.688	6.247

We can see that newly created impurity levels appear inside the gap, leading to the reduction of the bandgap as tabulated in Table 4. These reduced bandgaps are accommodated within the energy range of the solar light (1.5–2.8 eV), except ‘3/substituted’. By the partial DOS analysis given in Sec. ‘Computational Details’, we can identify the impurity levels mainly coming from Cu-3*d* and −4*s*. More multiplicity of the levels seen for ‘2/surface-attached’ and ‘3/substituted’ cases is because of the hybridization with O-2*p* state nearby the Cu site. For the ‘center-inserted’ case, the conduction band gets lower to touch the Fermi level, giving *n*-type behavior.

**Table 4:** The values of the highest occupied and the lowest unoccupied band as well as the electronic bandgap of the TiO_2_-NT structures.

Structure	Highest occupied band		Lowest unoccupied band		E _*g*_	
	Spin-up	Spin-down	Spin-up	Spin-down	Spin-up	Spin-down
Pristine (0,3) TiO_2_-NT	−2.82		0.34		3.16	
1/center-inserted	−2.16		0.44		2.60	
2/surface-attached	−1.92	−1.14	0.55	0.55	2.47	1.69
3/substituted	−2.64	−2.43	−1.33	−1.61	1.31	0.82
4/substituted-with-vac	−1.93	−2.47	0.34	−0.58	2.27	1.89
Bulk TiO_2_	0.05		2.63		2.58	

Units are all given in [eV].

From Fig. 3, we can immediately found that ‘1/center-inserted’ is the best candidate while in other cases impurity levels are located within the redox interval, [${\varepsilon }_{{{\rm{O}}}_{2}/{{\rm{H}}}_{2}{\rm{O}}},{\varepsilon }_{{{\rm{H}}}^{+}/{{\rm{H}}}_{2}}$], implying the possibility of the recombinations of excitations. Having such a candidate would support the capability of Cu-doped TiO_2_ nanotubes as a photocatalytic material^25^ to some extent, though the present prediction should be restricted within the limitation of the present computational model (tractable system size, and DFT-limited treatments of many-body effects for excitations).

**Figure 4:**
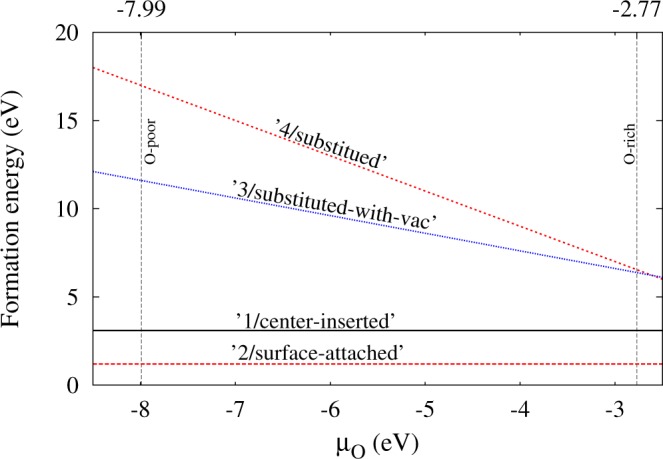
Calculated Formation energy of four cases considered for the Cu-doping model versus chemical potential of O (μ_O_) in the allowed interval.

While the ‘1/center-inserted’ case is shown to be the most promising for the desired reaction, the geometry is likely to be unstable from its energetics. The formation energies of the four cases, ${E}_{f}^{\mathrm{(1}-\mathrm{4)}}$, are evaluated as given in Sec.‘Computational Details’, shown in Fig. 4 as the dependence on the chemical potential of O (μ_O_). The values under the O-rich condition are also shown in Fig. 3. Compared with ‘2/surface-attached’ case, ‘1/center-inserted’ requires higher energy (+3.04 eV) to form the doped Cu located at the center. Though the higher formation energy would be required to form the geometry, we might expect the local stability for a Cu to be kept at the center against tiny displacement. We examined such possibility by looking at the energy variation when a Cu is displaced from the center, but the result could not support such possibility, giving the energy *increasing* corresponding to the local *maximum* energetically. It might be possible to attribute the observed transient property (photocatalyst activities decay in ∼100 [sec.])^25^ to this instability of a Cu at nanotube center. Furthermore, it would be likely to attribute their observed enhancement of photocatalytic activity to that realized by ‘1/center-inserted’ because a relevant experimental work^44^ did not found CuO peak in their XRD, implying that the doped Cu would be located not on the surface^44^.

## Conclusion

Structural and electronic properties of the anatase TiO_2_-NT were investigated for the pristine and the Cu-doped cases within GGA-PBE + *U* framework. The present computational modeling, especially the choice of the Hubbard *U* parameters for Ti and Cu, and the geometrical construction of the nanotube is confirmed to give fairly good agreements with available experimental data. To the extent of the tractable system size, the reported trend of the properties on the nanotube diameter is fairly well reproduced. We examined four possible models of the Cu-doped nanotube to investigate the capability for the photocatalytic activity, over the geometries with a Cu inserted to the center of tube, attached on the tube surface, and substitution of Ti site with and without an Oxygen vacancy. For all these cases, the introduced Cu gives impurity levels appearing in between the bandgap of the pristine case, leading effective reductions of the bandgap. Except the case with a Cu inserted to the center of the tube, the impurity levels are located within the redox interval, corresponding to the possibility of the recombinations of excitations. The case with the ‘center-inserted Cu’ satisfies the condition with *n*-type behavior that the conduction band and valance band edges located outside of the redox interval without such a possibility of recombinations, supporting the capability of such Cu-doped TiO_2_ nanotubes as a photocatalytic material^25^.

## Computational Details

For PW (plane wave)-DFT calculations, we used quantum ESPRESSO package^45^, with ultrasoft pseudopotentials^46^. The valence electronic states of Ti and O were considered as 3*s*, 3*p*, 4*s*, 3*d* and 2*s*, 2*p*, respectively. The pseudopotential of the Cu dopant was considered with the valence state, 3*d* and 4*s*. For the DFT + *U*^27^ scheme, we used GGA-PBE exchange-correlation functional^47^. The Hubbard parameter (*U*) for Ti is determined so that the calculated fundamental quantities (bandgap, bulk modulus *etc*.) can achieve reasonable coincidences to experimental values of the anatase TiO_2_ bulk. Convergence tests are performed to determine our final choice, *E*_cut_ = 40 Ry (cutoff energy for plane wave expansions) and 4 × 4 × 4 *k*-point grid. Cell and atomic positions are relaxed until the the forces on each atom get to the less than 1.05 meV/Å, which is feasible to be applied to all the following nanotube cases with a common condition. We adapted *U* = 4.2 eV for Ti taken from other works, which corrects the bandgap closer to the experimental value as shown in Table 1. Though the coincidence would be not so excellent within our GGA-PBE + *U*, we kept *U* untouched because it was adjustably obtained to give the correct splitting between occupied and unoccupied Ti-*d* states for O vacancies at the rutile TiO_2_ (110) surface^48^, and has been used to model both native *n*-type defects, and the (Nb,Ta)-doping in the anatase TiO_2_^49,50^. Moreover, the choice of *U* greater than 4 eV is reported to handle the electronic defects well^51^.

**Figure 5:**
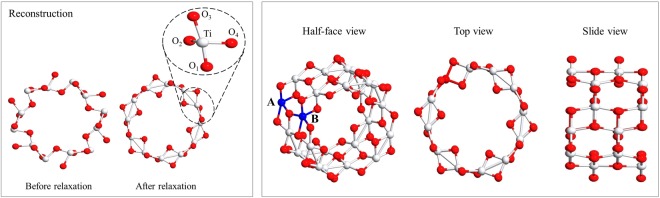
The geometrical reconstruction of (0,3) TiO_2_-NT from the bulk structure (left panel) and the 1 × 1 × 2 TiO_2_-NT unit cell from different view (right panel). The red and white spheres represent the O and Ti atoms, respectively. Ti atom of type A and B are in blue color.

For (0, *m*) nanotubes, we took the same *E*_c*ut*_ as that for the bulk because the same pseudopotentials are applied. To capture the property of an isolated tube by the plane wave framework, the tube is located inside a periodic box with an enough large spacing between parallel tubes by 30 Å, being confirmed to reduce spurious interactions between neighboring tubes within 10^−6^ eV. To make it tractable even when a Cu dopant is introduced, we took its unitcell as the multiple, 1 × 1 × 2, of the original unitcell for a bulk, namely twice larger supercell along the tube extending direction, which contains 72 atoms, as shown in Fig. 5. The Brillouin zone along the extending direction is discretized into four grid points to get the band dispersion. To make meaningful comparisons of the bandgap between bulk and tube cases, we carefully checked to choose the computational conditions so that they can be common to the both cases achieving the convergence with respect to the parameters.

It is observed that the geometrical reconstruction from the bulk structure makes it more symmetric with the reductions in the diameter and the thickness, as shown in Fig. 5 for instance with (0,3)-NT case. Corresponding numerical results are tabulated in the Table 2. It is found that the reduction of the diameter during the reconstruction is attributed to the reductions of the Ti-O bond lengths along the normal direction to the tube surface. This may be due to the change of the coordination number of O between bulk and nanotube due to the change of curvature^23^.

**Figure 6:**
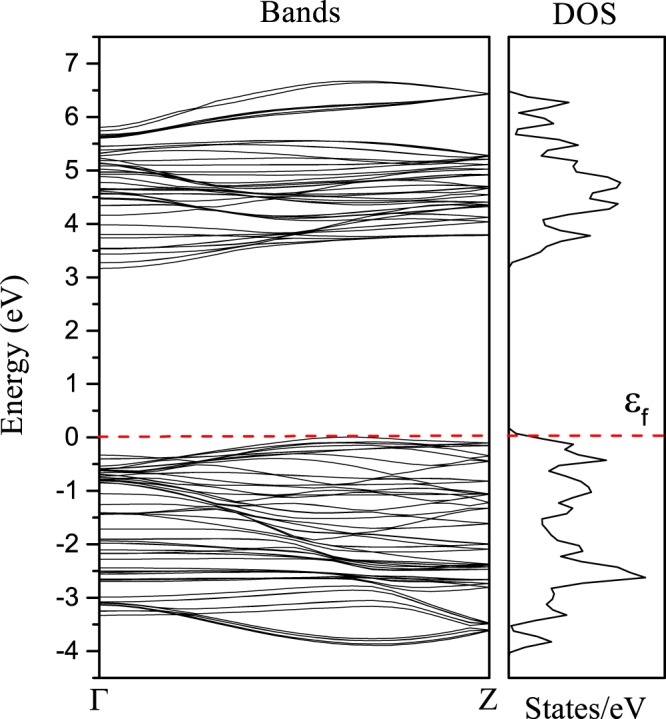
Electronnic band dispersion along the tube extending direction and total DOS of pristine (0,3) TiO_2_-NT.

The electronic band dispersion along the tube extending direction for (0,3)-nanotube as well as its total density of states (DOS) is given in Fig. 6. The indirect bandgap, 3.26 eV, is estimated being enhanced from that of bulk structure, 2.58 eV as given in Table 1. We note that the size of *m* = 3 is far smaller than the size of the practical TiO_2_-NTs, which outer diameters ranging 8–10 nm with an internal diameter being ∼5 nm^52^. When a Cu dopant is attached, however, the size of the computational model beyond *m* = 3 gets to be too large to be handled even using a large commercial supercomputer (*e*.*g*., Cray-XC40 we used here). This is not only because of the larger number of particles to be calculated (requiring more memory capacity), but also due to the exploding number of the freedom during the structural optimizations (even for *m* = 3, it took approximately one month for each case).

The formation energy of the nanotube tabulated in Table 2 is defined and evaluated as,

2 $${E}_{{\rm{f}}orm}=\frac{1}{n}({E}_{{\rm{total}}}-n{E}_{{\rm{bulk}}}),$$

as a normalized difference between the total energy of the system, *E*_total_, and that of the pristine bulk, *E*_bulk_ (per a TiO_2_ unit), where n is the number of the units included in the system.

**Figure 7:**
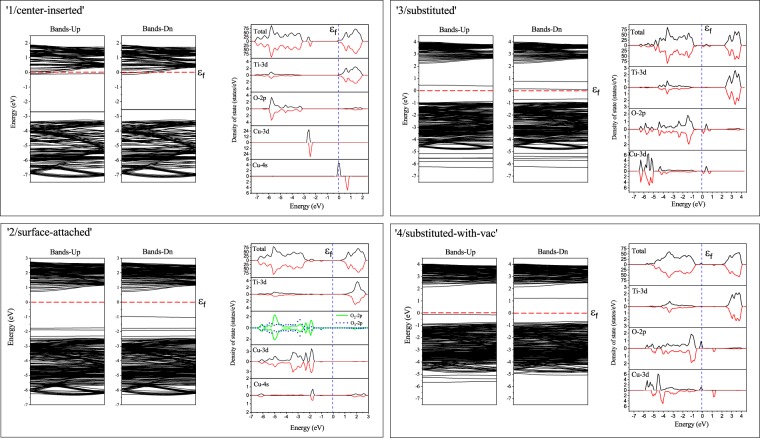
The electronic band structure along with the total and partial DOS of four cases considered for the Cu-doping model.

For Cu-doped model, we considered four cases, ‘1/center-inserted’, ‘2/surface-attached’, and ‘3,4/substituted (with-vac)’, as explained in Sec. ‘Result’. Figure 7 shows the electronic band structure, total and partial density of states (DOS) for ‘1/center-inserted’ and ‘2/surface-attached’ cases. For each case, we can see newly appearing sub-bands due to the introduced Cu, as discussed in Sec. ‘Results’. For both cases, we performed spin polarized calculations getting significant magnetizations, 0.58 *μ*_*B*_/cell on Cu (for ‘1/center-inserted’) and −1.00 *μ*_*B*_/cell on Ti (for ‘2/surface-attached’).

For the substitution case, there are two possibilities for a Ti site to be replaced by a Cu, marked as A and B in Fig. 5. Though they are different in their number of Ti-O bonds (four or five, respectively), we confirmed that the same structure is obtained after the relaxation regardless of each initial substitution. The schematic pictures showing geometrical change during the relaxation are given in Fig. 2, showing a slight compression of tube from top to bottom. The electronic band structure, total and partial DOS for the ‘3,4/substituted (with-vac)’ are also shown in Fig. 7. Similar to the ‘attached/inserted’ cases, we found several sub-bands appearing due to the introduced Cu and its hybridization with the neighboring Oxygen atoms when possible, as supported by the partial DOS identifying main ingredients. Spin polarized calculations conclude that the pronounced magnetizations appear on two O atoms neighboring to a Cu by 0.36 *μ*_*B*_/cell and 0.67 *μ*_*B*_/cell, respectively for ‘3/substituted’ case, while for ‘4/substituted-with-vac’, 0.49 *μ*_*B*_/cell on Cu and 0.17 *μ*_*B*_/cell on O, respectively.

To compose the band-lineup diagram (Fig. 3), we evaluated the work functions, shown in Table 3, using a scheme given in the literature^53^. We ought to note that the calculated work functions are overestimated when compared with experimental values (∼4.90 eV^54^) by 20%. Similar overestimations are also reported for the cases of carbon nanotube models when being smaller than 10 Å in diameters^55^, being attributed to the downshift of Fermi levels due to the curvature effect.

To discuss the stability of the doped structures, we also evaluated the formation energy to introduce a Cu dopant into a pristine TiO_2_-NT. To distinguish with *E*_form_ (formation energy to construct a NT), we shall use a different notation with the definition^56^,

3 $$\begin{array}{rcl}{E}_{f}^{\mathrm{(1,2)}} & = & {E}_{{\rm{tot}}}({\rm{Cu}}:{{\rm{Ti}}}_{24}{{\rm{O}}}_{48})-{E}_{{\rm{tot}}}({{\rm{Ti}}}_{24}{{\rm{O}}}_{48})-{\mu }_{{\rm{Cu}}},\\ {E}_{f}^{\mathrm{(3)}} & = & {E}_{{\rm{tot}}}({\rm{Cu}}:{{\rm{Ti}}}_{23}{{\rm{O}}}_{48})-{E}_{{\rm{tot}}}({{\rm{Ti}}}_{24}{{\rm{O}}}_{48})-{\mu }_{{\rm{Cu}}}+{\mu }_{{\rm{Ti}}},\\ {E}_{f}^{\mathrm{(4)}} & = & {E}_{{\rm{tot}}}({\rm{Cu}}:{{\rm{Ti}}}_{23}{{\rm{O}}}_{47})-{E}_{{\rm{tot}}}({{\rm{Ti}}}_{24}{{\rm{O}}}_{48})-{\mu }_{{\rm{Cu}}}+{\mu }_{{\rm{Ti}}}+{\mu }_{{\rm{O}}},\end{array}$$

where the upper suffix (*i* = 1–4) corresponds to four geometries such as ‘1/center-inserted’. Chemical potentials, μ_T*i*,Cu,O_, are basically determined by the experimental growth conditions. μ_Cu_ was considered as the energy of a Cu atom in the bulk structure. ${E}_{f}^{\mathrm{(1,2)}}$ is evaluated being +3.04 eV (+1.13 eV) for the ‘1/center-inserted’ (‘2/surface-attached’) case, as shown in Fig. 3. For the cases of ‘3,4/substituted (with-vac)’, the chemical potentials are determined so that they can satisfy the following criterion under thermodynamic equilibrium,

4 $${\rm{\Delta }}{H}_{f}({{\rm{T}}{\rm{i}}{\rm{O}}}_{2})={\mu }_{Ti}+2{\mu }_{O},$$

where Δ*H*_*f*_(TiO_2_) is the enthalphy of the formation of the bulk TiO_2_. The upper bounds of μ_O_ and μ_Ti_ can be obtained from the total energies of O molecule (*E*_*tot*_(O_2_)) and the Ti atom in its bulk structure (*E*_*tot*_(Ti _h*cp*_)), respectively. The allowed range of μ_O_ is therefore given as,

5 $$\frac{1}{2}({\rm{\Delta }}{H}_{f}({{\rm{TiO}}}_{2})+{E}_{{\rm{tot}}}({{\rm{O}}}_{2}))\le {\mu }_{O}\le \frac{1}{2}{E}_{{\rm{tot}}}({{\rm{O}}}_{2}),$$

corresponding to ‘O-poor’ and ‘O-rich’ conditions in the experimental growth processes. Our Δ*H*_*f*_(TiO_2_) is evaluated as −10.20 eV being in close agreement with the experimental value (−9.80 eV)^57^, measured at the growth condition. Corresponding to the variation of μ_O_, *E*_*f*_ for ‘3,4/substituted (with-vac)’ varies via relation, Eq. (4), as shown in Fig. 4. Formation energies for ‘3/substituted’ and ‘4/substituted-with-vac’ are evaluated to get 5.96 eV and 5.89 eV under the O-rich condition, respectively, showing little stabilization without the vacancy.

## Data Availability

All data generated or analysed during this study are included in this published article.
